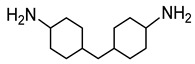# Ionic Liquids-Containing Silica Microcapsules: A Potential Tunable Platform for Shaping-Up Epoxy-Based Composite Materials?

**DOI:** 10.3390/nano10050881

**Published:** 2020-05-02

**Authors:** Ting Shi, Sébastien Livi, Jannick Duchet, Jean-François Gérard

**Affiliations:** UMR CNRS 5223 Ingénierie des Matériaux Polymères, Université de Lyon, INSA Lyon, 20, Avenue Albert Einstein, 69621 Villeurbanne, France; ting.shi@insa-lyon.fr (T.S.); jannick.duchet@insa-lyon.fr (J.D.); jean-francois.gerard@insa-lyon.fr (J.-F.G.)

**Keywords:** ionic liquid, silica, microcapsules, epoxy, network

## Abstract

In this work, silica microcapsules containing phosphonium ionic liquid (IL), denoted SiO_2_@IL, were successfully synthesized for the first time using the one step sol-gel method in IL/H_2_0 emulsion. The morphologies of the obtained micron-size microcapsules, including their diameter distribution, were characterized using dynamic light scattering (DLS), scanning electron microscopy (SEM), and transmission electron microscopy (TEM). The thermal behavior of these microcapsules and the mass fraction of the encapsulated IL in the silica microcapsules were determined using thermogravimetric analysis, showing an excellent thermal stability (up to 220 °C) and highlighting that an amount of 20 wt.% of IL is contained in the silica microcapsules. In a second step, SiO_2_@IL microcapsules (1 wt.%) were dispersed into epoxy-amine networks to provide proof of concept of the ability of such microcapsules to act as healing agents as microcracks propagate into the epoxy networks.

## 1. Introduction

According to the literature on fiber-based polymer matrix composites (PMC) based on thermoset matrices, it is well-known that when such structural materials are damaged, various strategies could be deployed to extend their lifetime. Nevertheless, among these, the type of damage must also be taken in consideration in order to propose relevant repair strategies. For example, if the damage is accessible, such as matrix cracking, the simplest approach consists of a direct injection of an epoxy reactive system in the crack space, but if a fiber fracture occurs, a recovery of the fiber remains impossible. Thus, self-healing strategies have been mainly focused on polymer matrix healing [1]. It should also be pointed out that these approaches to the repair of thermosetting matrices of composite materials cannot be compared with the numerous works that have been developed in recent years on reversible elastomer networks for which the intrinsic molecular mobility (since they are considered at a temperature higher than their glass transition temperature (T_g_)) allows healing without temperature stimulus. The same applies to most of the work carried out on vitrimers, which do not have sufficient glass transition temperatures for composite material applications subject to high operating temperatures. Thus, for high T_g_ polymer networks used as matrices in composite materials, the reported strategies relate to (i) extrinsic self-healing such as embedding in microcapsules or hollow tubes (which can be disposed as a microvascular network) containing a liquid phase, i.e., the healing agent [2,3], in order to continuously deliver the healing agent into cracks via the capillary driving force and (ii) intrinsic self-healing such as the introduction of reversible bonds into polymer matrix architecture, such as (multi-) hydrogen bonds, or host-guest structures, such as metal ligands, or the use of thermally reversible Diels-Alder (DA) bonds, which can heal the micro-cracks as many times as required from a thermal heating and cooling cycle which allows reforming of the DA bonds between furan and maleimide groups [4,5]. Our group has also created carbon fiber/epoxy microcomposites with thermo-reversible interfaces by incorporating compounds with furan and maleimide groups through DA reaction [6,7].

In the last decade, ionic liquids (ILs) have emerged as new components within polymer-based materials for a wide range of applications. Due to their unique set of physic-chemical properties as well as their multitude of chemical structures, ILs allow promising new pathways to design new (multi)functional objects and materials. Indeed, the inclusion of ionic liquids in polymer science has been very rapid in recent years by considering them as solvents or as interfacial agents in polymer blends or organic-inorganic hybrid materials up to their use over the time as functional additives to polymer matrices. More recently, ILs have generated a growing interest in the field of thermosetting polymers such as epoxies or cyanate esters [8] or they can be applied for designing new IL-epoxy networks [9]. Applied to epoxy networks, ILs offer new pathways for designing novel networks by considering IL as unreactive or reactive functional additives. Currently, numerous works described in the literature relate the key role of ionic liquids as functional components of epoxy prepolymers as they could act as catalysts of epoxy-amine reactions or as initiators of an anionic polymerization [10,11], dispersant aids of nanoparticles in epoxy reactive systems and resulting networks [12], and compatibilizer in epoxy-thermoplastic blends. In addition to all of those studies dedicated to the addition of IL to epoxy reactive systems for conventional purposes, Sanes et al. [13] have recently investigated the influence of ILs on the tribological performances of epoxy-amine network surfaces as these are known to be interesting lubricating agents [14]. In their study, these authors prepared reactive systems based on an epoxy prepolymer cured with a mixture of diamines which was combined with different amounts (from 7 wt.% to 12 wt.%) of imidazolium-IL, i.e., 1-octyl-3-methylimidazolium tetrafluoroborate, in order to evaluate the self-healing ability of the resulting epoxy surfaces [15]. Still in the field of tribological applications, a Chinese team has developed an innovative in situ self-lubrication route based on the preparation of polymer microcapsules containing ionic liquids for epoxy-amine thermoset matrices. In fact, Li et al. [16] have designed polysulfone (PSU) microcapsules containing imidazolium-IL in order to reduce the frictional coefficients between a metal surface and an epoxy matrix. Thus, encapsulated IL/PSU microcapsules of 128 µm diameter were prepared. The influence of the microcapsule content (from 0 to 30 wt.%) on the tribological performances was investigated. Even if this approach may be difficult to apply due to the IL viscosity, the bipolar structure, and the ionic interactions, such a route appears to be promising for the design of self-healable polymer materials. Other authors such as Luo et al. have reported a method to trap imidazole-IL in a polymer shell using microencapsulation to propose a new solvent extraction system [17]. Inorganic components can be good candidates for designing the shell of such IL containers. More specifically, silica was considered for preparing nano/microcapsules for controlled release applications [18] thanks to its good mechanical properties [19] and biocompatibility [20,21]. Recently, Abu-Reziq et al. [22] have used lignosulfonic acid to stabilize ionic liquid in water in order to prepare a silica shell according to an interfacial sol-gel process. The obtained microcapsules showed good performances as heterogeneous catalysts. Yang et al. have used the same ionic liquid as core and a natural emulsifier, i.e., gelatin, to obtain microcapsules which can promote improved tribological behavior of polyurethane composite materials.

Nevertheless, there are no publications reporting works dedicated to the encapsulation of phosphonium ionic liquids in a silica shell or IL microcapsules as functional additive in epoxy networks. Thus, inspired by self-healing IL-epoxy composites and encapsulation of IL, this work aims to design through a simple route a new type of phosphonium ionic liquid core/silica shell microcapsule prepared using sol-gel chemistry in microemulsion, in which a silica shell provides perfect stability of the IL until the breakage of the microcapsules. For the first step, the synthetic method and basic characteristics of microcapsules (denoted SiO_2_@IL) such as the morphology, size distribution, chemical structure, and thermal behavior are carefully investigated before being considered as functional additives in epoxy matrix-based composite materials. What is more, as a first proof of concept, such ionic liquid containing microcapsules will be introduced in epoxy-amine networks to investigate their contribution to the basic properties of the epoxy-based networks, while other properties need to be further studied.

## 2. Experimental 

### 2.1. Materials

Sodium dodecyl sulfate (SDS), cetyltrimethylammonium bromide (CTAB), tetraethyl orthosilicate (TEOS), and ammonium hydroxide solution (NH_3_·H_2_O) were purchased from Sigma-Aldrich Co (St. Louis, MO, USA). The phosphonium ionic liquid based on trihexyl(tetradecyl) phosphonium cation combined with bis(2,4,4-trimethylpentyl) phosphinate counter anion, denoted Cyphos IL 104, was provided by Solvay, Inc (Niagara Falls, Canada). Bisphenol A diglycidyl ether (DGEBA, D.E.R. 332) and 4,4′-methylenebis(cyclohexylamine) (PACM) were also both supplied from Sigma-Aldrich Co (St. Louis, MO, USA). Acetone was purchased form Carlo ERBA Reagents Co (Paris, France). The chemicals were used as received without any purification. All the chemical structures are shown in Table 1.

### 2.2. Synthesis of Core-Shell SiO_2_@IL Microcapsules

**Figure 1:**
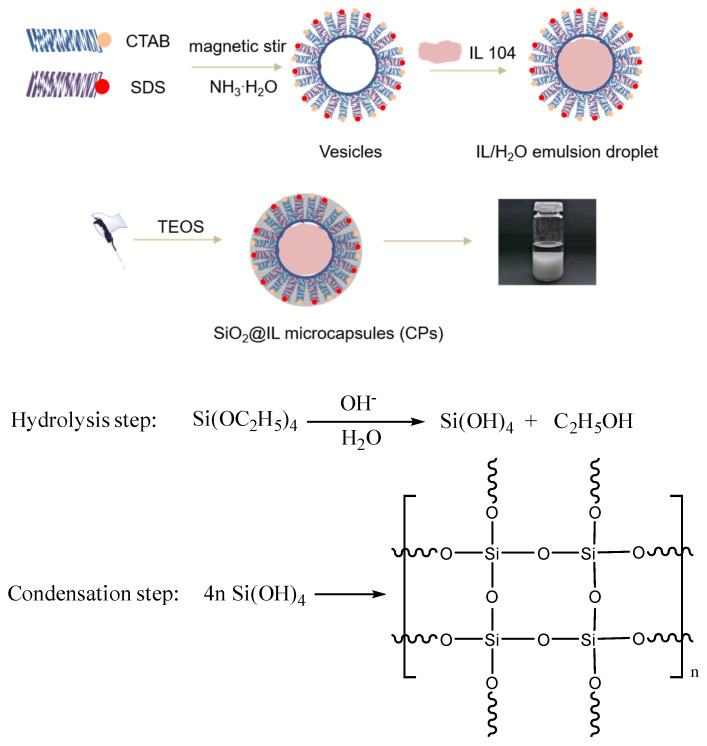
Different steps of the synthesis of silica microcapsules containing phosphonium ionic liquid (SiO_2_@IL).

Figure 1 illustrates the synthesis route to prepare the microcapsules, denoted SiO_2_@IL CPs. In a first step, a cationic surfactant, CTAB, and an anionic surfactant, SDS, were added in water until they were fully dissolved. The electrostatic interactions between the positively and negatively charged head groups of CTAB and SDS drove these molecules to form vesicles in water [15,16]. After adding IL 104, the long alkyl chains of IL 104 helped them to diffuse into the vesicles and finally form ionic liquid in water (IL/H_2_O) emulsion under stirring. To form the silica shell, tetraethoxysilane (TEOS) was chosen as precursor and ammonium hydroxide was added. Under these conditions, TEOS was transferred at the IL/water interface to undergo hydrolysis. The hydrogen bonding interactions of so-formed silanols lead to gelation and further condensation reactions at the IL/H_2_O interface of the emulsified droplets in order to form the silica-like shell. In a final step, the white precipitate was collected using centrifugation and dried at 60 °C for 12 h to be further characterized.

With more details, 0.20 g SDS and 0.40 g CTAB were dissolved in 54 mL deionized water and 1.00 mL NH_3_·H_2_O was added to the mixture. Then, the mixture underwent magnetic stirring and was kept at 68 °C for 1.5 h in order to obtain a homogeneous solution. Subsequently, 1.00 g of IL 104 was added in the solution and vigorously stirred at 68 °C for an additional time of 5 h until obtaining a milky solution of ionic liquid as a water (IL/H_2_O) emulsion. After that, 1.50 g TEOS was dripped slowly into the (IL/H_2_O) emulsion to perform the TEOS sol-gel reaction to form the silica shell. The sol-gel process was kept at 68 °C for 2 h and then at 80 °C for 2 h. Finally, a white precipitate was collected using centrifugation and washed three times with deionized water before being dried at 60 °C for 24 h.

As comparative, hollow silica without IL was also synthesized as follows [23]: 0.0665 g SDS and 0.1335 g CTAB were dissolved in 27 mL deionized water. The, 0.5 mL NH_3_·H_2_O was added to the mixture. After the SDS and CTAB were fully dissolved, 2.0 mL TEOS was dropwise added and stirred at 68 °C for 2 h and 80 °C for 2 h. Finally, white precipitate was separated from the reaction mixture and washed three times with water. 

### 2.3. Preparation of Epoxy-Amine Reactive Systems and Epoxy-Amine Containing SiO_2_@IL CPs Networks

**Figure 2:**
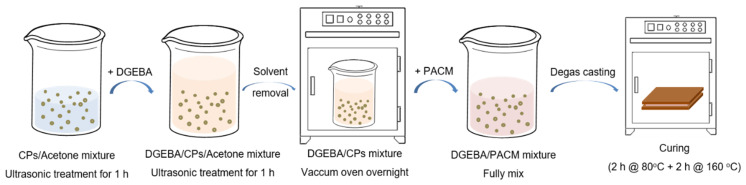
Schematic preparation procedure of SiO_2_@IL CPs/epoxy-amine networks.

The preparation procedure of the ionic liquid core/silica shell microcapsules (SiO_2_@IL CPs)/epoxy-amine networks is described in Figure 2. SiO_2_@IL CPs were added in a given amount of acetone and the mixture was sonicated for 1 h to disperse the microcapsules uniformly in acetone. Then, the corresponding amount of epoxy prepolymer (DGEBA, D.E.R 332) was added in the mixture and kept under ultrasonic stirring for an additional hour. After, the mixture was put in a vacuum oven at 70 °C overnight to remove all of the solvent. Then, according to the stoichiometric ratio between the number of the epoxy groups and the functionality of the 4,4’-diaminodicyclohexylmethane (PACM), the corresponding amount of PACM was added to the epoxy prepolymer and mixed under mechanical stirring. In a final step, the CPs/epoxy-amine mixture was degassed under vacuum for 10 min, cured as 5 mm-thick films at 80 °C for 2 h, and post cured at 160 °C for 2 h.

### 2.4. Characterization

A transmission electron microscope (TEM) equipped with energy dispersive spectroscopy (EDS) was used with Phillips CM 120 (Waltham, MA, USA) microscopy operating at an accelerating voltage of 120 kV. The cured epoxies were cut using ultramicrotomy with a cross section thickness of about 60 nm. Scanning electron microscope (SEM) was performed using a ZEISS MERLIN COMPACT (Munich, Germany) microscope operating at an accelerate voltage of 1 kV to analyze the morphology of the microcapsules. 

Thermogravimetric analysis (TGA) of the SiO_2_@IL CPs and the corresponding epoxy-amine networks was carried out using a Q500 thermogravimetric analyzer (TA Co. Ltd., New Castle, DE, USA) from 30 to 700 °C for a heating rate of 20 K min^−1^ under nitrogen atmosphere.

Dynamic light scattering (DLS) performed on a Nano-Zeta Sizer Nano Series instrument, and a ZEN3600 (Malvern Instruments, Malvern, UK) was used to determine the size distribution of the SiO_2_@IL microcapsules. The sample was diluted in pure distilled water. The dilution was optimized for each sample in order to obtain an accurate signal-to-noise ratio.

The chemical structures of the reagents and microcapsules were characterized using Fourier-transform infrared spectroscopy (FTIR) using a Thermo Scientific Nicolet iS10 Spectrometer (Waltham, MA, USA) in transmission mode (64 scans, resolution 4 cm^−1^) with potassium bromide pellets.

Elemental analysis was carried out by CREALINS Co. using an inductively coupled plasma-atomic emission spectrometry (ICP-AES) instrument from Thermofisher Instrument. 

Differential scanning calorimetry (DSC) measurements performed on epoxy composites were carried out using a Q10 TA Co. Ltd., New Castle, DE, USA)) from 20 to 250 °C at a heating rate of 10 K·min^−1^ under a nitrogen flow of 50 mL·min^−1^.

## 3. Results and Discussion

**Figure 3:**
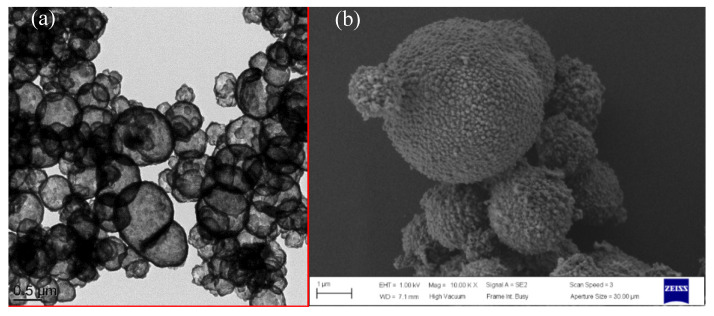
TEM (**a**) and SEM (**b**) micrographs of SiO_2_@IL microcapsules.

Transmission electron microscopy (TEM) and scanning electron microscopy (SEM) are powerful tools to determine the morphology distribution and surface morphology of silica microcapsules [24,25] or other micro/nanostructures from the microscopic viewpoint [26,27]. Figure 3 shows the TEM and SEM images of the SiO_2_@IL microcapsules. They reveal the core-shell structure and the roughness of the obtained microcapsules. The TEM micrograph in Figure 3a proves the successful synthesis of silica microcapsules having sizes from 0.5 to 2 μm. This distribution of particle sizes of the microcapsules can be explained by the synthesis conditions of the protocol involving a sol-gel process in O/W emulsion medium. The dispersed oil droplets in the emulsion are always in constant motion. As a consequence, if the interfacial membrane of the oil droplets in the emulsion breaks during collision, two droplets can coalesce to form larger size droplets. [28]. Nevertheless, the presence of an emulsifier, to stay at a given temperature under continuous stirring, is required to keep the emulsion stable. However, in different locations of the emulsion, the droplet is subjected to different shear forces leading to a broad droplet size distribution [29,30]. In addition, the shell thickness of the microcapsules is estimated from image analysis to be 100 nm. On the SEM micrographs, it is also worth noting the roughness of the silica microcapsules, highlighting the formation of colloidal silica nanoparticles on their surface. In addition, energy-dispersive X-ray spectroscopy allowed to determine the location of the phosphonium ionic liquid in the silica shell microcapsules. Compared to the carbon-film-supported copper grids used as reference substrate, silicon and phosphorus-rich compounds can be clearly distinguished in the microcapsules confirming that the phosphonium IL is well encapsulated in the silica shell. 

**Figure 4:**
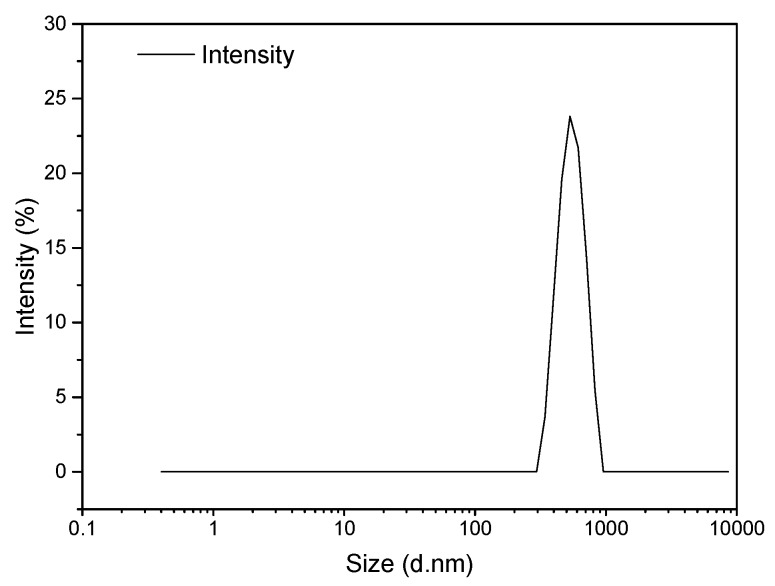
Dynamic light scattering (DLS) pattern of SiO_2_@IL microcapsules.

Dynamic light scattering (DLS) was considered to quantify the microcapsule size distribution already observed using electron microscopy. The DLS spectrum presented in Figure 4 evidences a relatively narrow distribution of particle sizes with a microcapsule Z-average size of close to 720 nm, which is within agreement with the conclusions from TEM and SEM analyses. As mentioned previously, the polydispersity of microcapsule sizes is associated with the dynamic character of IL/H_2_O emulsion droplets [29].

**Figure 5:**
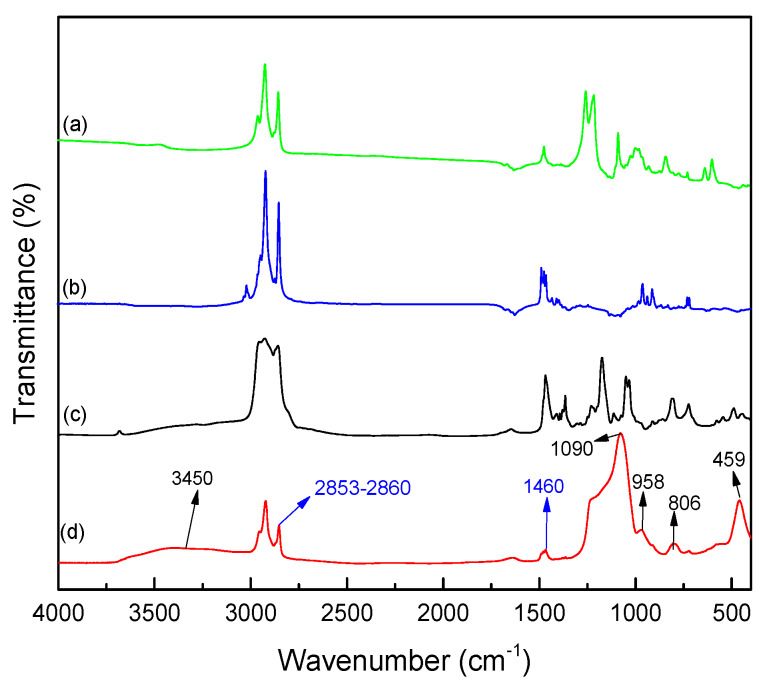
FT-IR spectrum of (**a**) sodium dodecyl sulfate (SDS), (**b**) cetyltrimethylammonium bromide (CTAB), (**c**) IL 104 and (**d**) SiO_2_@IL.

In addition, the chemical structure of the microcapsules was characterized using FTIR spectroscopy considering the IR spectra of the various components. The infrared spectra of the microcapsules, IL 104, and surfactants, i.e., CTAB and SDS, are presented in Figure 5. As can be seen from the spectra, the strong and wide absorption band at 1090 cm^−1^, which corresponds to anti-symmetric stretching vibrations of Si–O–Si bonds as well the absorption bands at 806 cm^−1^ and 459 cm^−1^ which are assigned to symmetric stretching vibration and bending vibration of Si–O bonds [31], are a proof of the existence of the SiO_2_-rich shell. Moreover, the stretching vibration of C–H bonds (2853–2860 cm^−1^) and bending vibration of –CH_2_– groups (1460 cm^−1^) come from the alkyl chains resulting from both the ligant in IL and the hydrophobic parts of the surfactants. Compared to the strong absorption peaks of silica shell, these relatively weak absorption peaks corresponding to aliphatic chains can be additional evidence to demonstrate that the IL is well encapsulated. In addition, the similar absorption of the IL and microcapsules confirms the good encapsulation of the IL. In order to indicate the mass fraction of IL 104 and silica shell, further analytical characterizations should be carried out.

**Figure 6:**
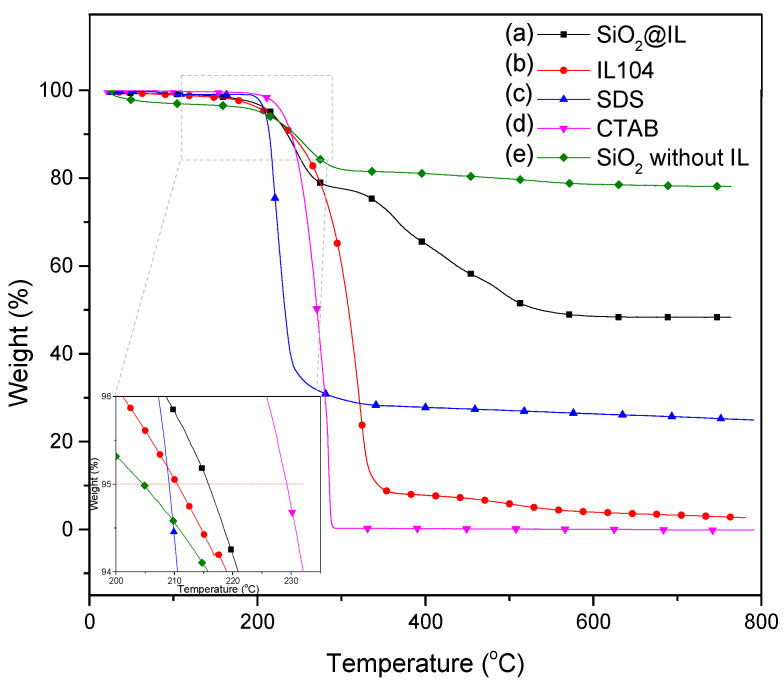
TGA curves of (**a**) SiO_2_@IL, (**b**) IL 104, (**c**) SDS, (**d**) CTAB, and (**e**) SiO_2_ without IL (heating ramp: 10 K min^−1^).

As we now knew that the ionic liquid is well encapsulated in the silica shell of the microcapsules, a thermogravimetric analysis (TGA) was carried out to quantify the amount of the trapped phosphonium IL as well as to characterize the thermal stability of this new type of microcapsule. The TGA traces of the microcapsules, IL 104, and the surfactants are presented in Figure 6. The onset decomposition temperature was defined as the temperature at which a 5 wt.% loss is observed. This decomposition temperature was found to be 208, 209, and 231 °C for IL 104, SDS, and CTAB, respectively. For SiO_2_@IL, the onset decomposition temperature is slightly higher than for neat IL (216 °C) due to the protection of the ionic liquid by the silica shell and the resistance of the latter to the internal pressure. In order to better understand the thermogravimetric behavior, we used SiO_2_ without IL as a reference, which was synthesized using the same procedure as the SiO_2_@IL microcapsules, the only difference was the lack of IL. The first degradation undergone by SiO_2_@IL and SiO_2_ without IL is mainly due to the decomposition of surfactants. Furthermore, the comparison with SiO_2_ without IL highlights an additional degradation which can be assigned to the main degradation of IL. Regarding the complex degradation process, it was found that the total weight loss of the microcapsules is about 51 wt.%. In addition, from the elemental mass fractions of Si and P, i.e., 19.1% and 1.5% respectively, the corresponding SiO_2_ and IL 104 contents were estimated in the microcapsules to be 41 wt.% of silica and 20 wt.% IL 104, while the other weight loss can be attributed to the degradation of surfactants. The calculated mass fraction of the silica is in agreement with the residual weight fraction measured using TGA after complete degradation. As a comparison, 66 wt.% SiO_2_ and 31 wt.% IL based microcapsules were prepared by Weiss [22] and Zhang [32] for microcapsules containing imidazolium ionic liquids. 

To sum up, we have successfully synthesized microcapsules with a silica shell containing phosphorous ionic liquid. It has been proved that this new type of microcapsule has a spherical morphology and offers a high thermal stability in comparison to IL alone. Moreover, the trapped ionic liquids could be considered now as a tunable additive for the development of functional epoxy-based composite materials. Up to now, only very few works have reported the encapsulation of imidazolium ionic liquid using a polymer shell. For example, thermoplastics like polysulfone [33] or poly(tetrafluoroethylene) (PTFE) were considered as shells for microreservoirs containing ILs [34]. Thus, according to the superior properties of SiO_2_ compared to polymers, ionic liquid encapsulated in silica microcapsules could be promising as new and relevant functional agents microparticles for thermosets networks. Such microcapsules could play two roles either as toughening agents or as functional agents like self-healing agents. 

From these results, we propose to add these ionic liquid functionalized microcapsules in a thermoset network to investigate the morphology of such modified networks as well as their final properties. It is obvious that according to the high versatility of available ionic liquids as well as the low density of the SiO_2_@IL CPs compared to usual inorganic nano/micro particles, these new SiO_2_@IL microcapsules could offer a great and tunable potential for thermoset matrices such as epoxies in polymer composite materials as functional additives. In order to investigate the potential of such microcapsules in thermoset-based polymer composites, a commonly used epoxy network was chosen as matrix. The well-known bisphenol A diglycidyl ether combined with 4,4′-methylenebis(cyclohexylamine) (PACM) as a comonomer with a stoichiometric ratio of epoxy-to-amino hydrogen equal to 1 was selected as reactive system due to the easy handling of such formulation according to the liquid state of the reactants at room temperature and the high T_g_ of the fully cured network. Moreover, a small amount of microcapsules was introduced in the epoxy-amine system to evaluate the resistance of these microcapsules during the network processing, i.e., mixing and curing steps. In a first step, the morphology of the microcapsules in the cured network and the thermal properties of epoxy composites were investigated. 

**Figure 7:**
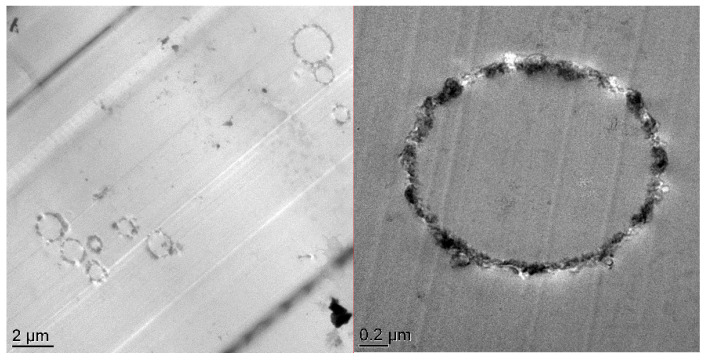
TEM micrographs of SiO_2_@IL microcapsules embedded in an epoxy-amine network at different magnifications.

To observe the morphology of the microcapsules in the epoxy-amine networks as well as the dispersion of the microcapsules in the matrix, ultrathin slices of SiO_2_@IL-modified epoxy microcomposites were observed using TEM, as shown in Figure 7. The silica shell of about 100 nm thickness of the microcapsules can be clearly distinguished. These results clearly confirm the SEM images of the pristine silica microcapsules (see Figure 3a). Moreover, the relatively good dispersion of microcapsules at the concentration of 1 wt.%, may be promising for the physical properties of microcomposites as the dispersion state plays a crucial role [19]. 

**Figure 8:**
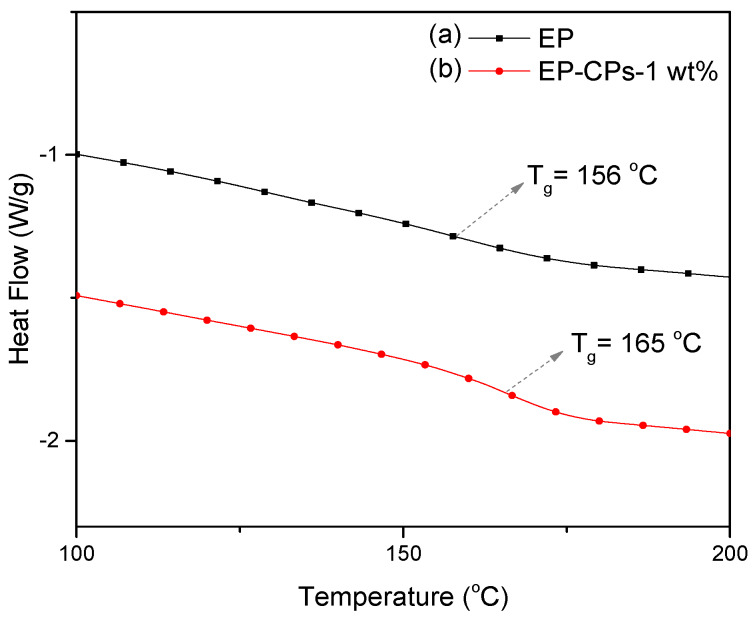
Differential scanning calorimetry (DSC) traces of (**a**) epoxy-amine network (EP) and (**b**) microcomposite containing 1 wt.% of SiO_2_@IL microcapsules (EP-CPs-1 wt.%) (heating: 10 K min^−1^).

**Figure 9:**
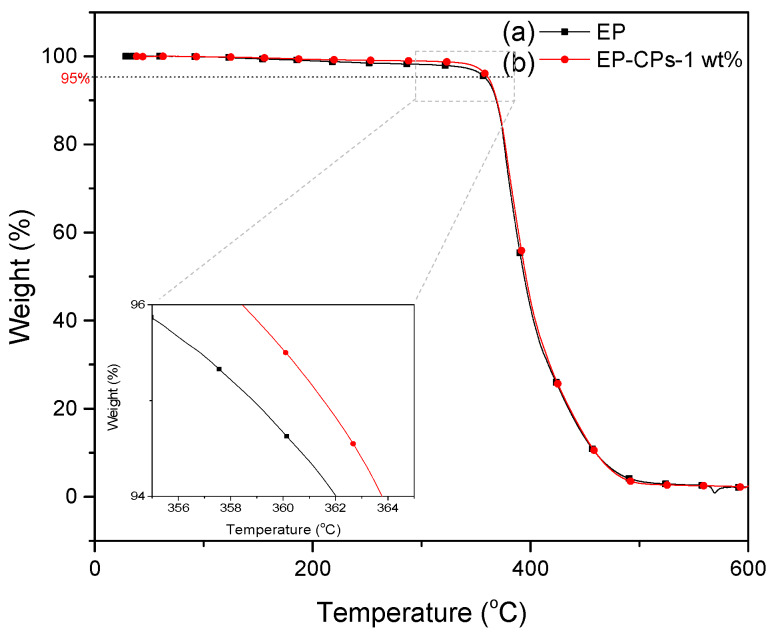
TGA traces of (**a**) EP and (**b**) EP-CPs-1 wt.% materials (heating rate: 10 K.min^−1^).

The thermal behavior of SiO_2_@IL microcapsules/epoxy-amine microcomposites was investigated using DSC and TGA methods (Figure 8 and Figure 9). As shown in Figure 8, the neat epoxy-amine network (EP) and the microcomposite containing 1 wt.% of SiO_2_@IL microcapsules (EP-CPs-1 wt.%) display a glass transition temperature, T_g_, of 156 and 165 °C, respectively. This increase of T_g_ may be attributed to the chemical interactions occurring at the silica shell/epoxy network interface, which leads to a reduction in molecular mobility. The presence of silanol groups on the silica shell surface may form hydrogen bonds with the polar species of the epoxy network such as the hydroxyether groups formed by the reaction between glycidyl functions of DGEBA prepolymer and amines [35]. Previous works showed that the addition of sub-micron silica as fillers in epoxy networks improved the mechanical property of epoxy composites both in the glassy and rubbery states but the performance clearly depended on the filler dispersion [36]. The physicochemical analysis of the interfaces and the characterization of the microstructure of the silica/epoxy networks highlighted that the covalent bonding between the silica particles and the epoxy is responsible for the performance of the final epoxy composites [37].

Let’s now consider the impact of microcapsules on the thermal degradation of the epoxy-amine networks (EP) under nitrogen atmosphere. Similar decomposition behaviors are observed under nitrogen atmosphere. In fact, the decomposition temperatures (considered for 5% weight loss) of EP and EP-CPs-1 wt.% are 358 and 361 °C. Because of the low content of the microcapsules, there is no difference of the residual weight for the epoxy-amine network without or with microcapsules.

## 4. Conclusions

Ionic liquid (IL) core/silica shell microcapsules were successfully synthesized from the one step sol-gel hydrolysis and condensation reactions of TEOS within an IL/H_2_O emulsion. TEM and SEM microscopies clearly evidence the core-shell structure and spherical shape of the synthesized SiO_2_@IL microcapsules, i.e., microreservoirs, with a SiO_2_-like shell and IL trapped as core. EDX (performed under TEM) and FTIR spectroscopies have proved that the phosphonium-based ionic liquid is well encapsulated in the silica shell. Thermogravimetric and elemental analyses also show that the mass fraction of the ionic liquid (IL) and silica shell are close to 20 wt.% and 41 wt.%, respectively. Moreover, the microcapsules show very good thermal stability, which allows their use for being inserted in a curable system such as an epoxy-amine reactive system for processing microcomposites. Consequently, this study paves the way for the implementation of such ionic liquid core/silica shell microcapsules as promising additives in epoxy networks to promote improved mechanical properties with a potential ability for self-healing performances.

## Figures and Tables

**Table 1:** Materials and their chemical structures.

Materials and Abbreviations	Chemical Formula
Ionic liquid Tetradecyl(trihexyl)phosphonium bis(2,4,4-trimethylpentyl)phosphinate (IL 104)	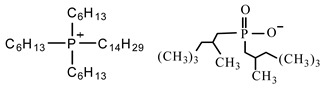
Epoxy resin Bisphenol A diglycidyl ether (DGEBA D.E.R. 332 n=0.03, where n refers to number of repeating units, $n=\frac{M_{W}-348}{315}$)	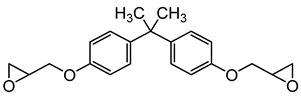
4,4′-methylenebis(cyclohexylamine) (PACM)